# Development of a Ground-Based Hyperspectral Remote Sensing System for High-Frequency Monitoring of Riverine Organic Carbon

**DOI:** 10.3390/s26154751

**Published:** 2026-07-27

**Authors:** Wei Gao, Xianqiang He, Xuan Zhang, Xuchen Jin, Fang Gong

**Affiliations:** 1 College of Oceanography, Hohai University, Nanjing 210098, China; 231311040029@hhu.edu.cn; 2 State Key Laboratory of Satellite Ocean Environment Dynamics, Second Institute of Oceanography, Ministry of Natural Resources, Hangzhou 310012, China; 3 Key Laboratory of Ocean Observation and Information of Hainan Province, Sanya 572000, China

**Keywords:** ground-based hyperspectral remote sensing system, dissolved organic carbon, particulate organic carbon, high-frequency observation

## Abstract

Traditional approaches for monitoring aquatic organic carbon, such as satellite remote sensing and automated underwater sensors, are often constrained by limited temporal resolution, data gaps under cloudy conditions, maintenance requirements, and cost-effectiveness. To overcome these limitations, we developed and field-demonstrated a ground-based hyperspectral remote sensing system (GHRSS) for continuous, high-frequency monitoring of dissolved organic carbon (DOC) and particulate organic carbon (POC). The system is based on the above-water method and integrates three miniature hyperspectral spectrometers to measure water-surface radiance, sky radiance, and downwelling irradiance for deriving hyperspectral remote sensing reflectance ($R_{rs}$). The spectrometers cover 400–900 nm with a spectral resolution of 1 nm and support a minimum sampling interval of 10 s. The GHRSS also integrates solar power supply, 4G communication, and a microcomputer, enabling autonomous long-term deployment and wireless data transmission. Based on the GHRSS, retrieval models for DOC and POC were developed and validated using 90 paired in situ measurements collected from the Cao’e River. Empirical and machine learning methods were applied to retrieve DOC and POC from the measured $R_{rs}$ data. The empirical models showed limited retrieval performance, whereas partial least squares regression (PLSR) and support vector regression (SVR) substantially improved model accuracy. Among all models, SVR achieved the best performance on the independent test set, with $R^{2}=0.979$, RMSE = 0.031 mg/L, and MAE = 0.024 mg/L for DOC and $R^{2}=0.960$, RMSE = 0.152 mg/L, and MAE = 0.066 mg/L for POC. Using the optimal SVR models, minute-scale time series of DOC and POC were reconstructed from the GHRSS observations. The results revealed pronounced sub-daily variability in both parameters, with DOC varying relatively smoothly, whereas POC exhibited stronger short-term fluctuations and more rapid responses to hydrodynamic changes. These findings demonstrate that the GHRSS, combined with machine learning models, provides an effective and practical approach for continuous, high-frequency monitoring of riverine organic carbon dynamics.

## 1. Introduction

Aquatic ecosystems play an important role in the global carbon cycle by storing, transforming, and transporting organic carbon, thereby influencing regional carbon budgets and climate feedbacks [1,2,3]. In natural waters, organic carbon is commonly quantified as dissolved organic carbon (DOC) and particulate organic carbon (POC) [4,5]. These two reservoirs vary in their sources, transport pathways, and biogeochemical fates, collectively regulating ecosystem metabolism and carbon exchange among land, water, and the atmosphere [6,7,8]. DOC represents the largest organic carbon pool in aquatic environments and is closely associated with microbial processes [4,7]. Due to its dissolved form, DOC is primarily utilized by bacteria and archaea through the microbial loop, during which part of the carbon is respired as CO_2_ and released to the atmosphere [2,9]. Inland waters, including rivers, lakes, and reservoirs, therefore act as active sources of atmospheric CO_2_ [1,3]. In addition, a fraction of DOC can be transported over long distances within river networks, contributing significantly to lateral carbon fluxes from land to ocean [10,11]. In contrast, POC plays a dominant role in particulate transport and vertical carbon flux. It supports aquatic food webs and contributes to long-term carbon sequestration through sedimentation, forming a key component of the biological pump [12,13,14]. DOC and POC are dynamically linked through physical, chemical, and biological processes, particularly in rivers and estuaries where hydrodynamic conditions and human activities strongly influence organic carbon transformation and export [6,15,16].

Despite their importance, high-frequency monitoring of DOC and POC concentrations and tracking their short-term variations remain challenging [17,18,19]. At present, the traditional methods for determining DOC and POC involve field water collections and then bringing them back to the laboratory for measurement. This approach can provide more accurate information, however, it is time-consuming, laborious, and lagging because water sampling and laboratory analysis require considerable labor and time [20,21]. In addition, the approach relies on manual water sampling at preset sampling sites and at regular (biweekly or monthly) intervals or via short-term ship cruises and thus can provide only limited data on DOC and POC dynamics [17,18]. As a result, rapid changes driven by tidal forcing, hydrological regulation, rainfall events, or diurnal biological activity are often missed [17,18,19]. Furthermore, in the past two decades, satellite remote sensing has been widely used to estimate water quality parameters due to its advantages of large scale, long duration, and periodicity and cost-efficiency [22,23,24]. However, its application is constrained by revisit frequency, cloud contamination, and limited data availability under complex weather conditions [25,26]. Although DOC and POC are not optically active constituents, indirect retrieval using empirical or machine learning models has become possible through their coupling with optically active substances, such as colored dissolved organic matter (CDOM) and suspended particles [27,28]. Nevertheless, satellite observations are generally insufficient to capture sub-daily DOC and POC dynamics. The flexible and mobile unmanned aerial vehicle (UAV) hyperspectral remote sensing has partially made up for the above defects [29], but limited hover time and complex weather hinder continuous monitoring. There are already several in situ optical systems for real-time monitoring of water quality in inland waters. Representative instruments frequently used in published field studies include submersible FDOM fluorometers and submersible spectrophotometers [30]. However, apart from the high cost, these underwater sensors are disturbed by temperature and turbidity and require regular maintenance (biweekly and monthly) due to the occurrence of biofouling. Moreover, data loss occurs occasionally when the concentration exceeds the measurement range [31].

Consequently, ground-based above-water hyperspectral remote sensing provides a promising alternative for continuous water quality monitoring. By measuring water-leaving radiance and downwelling irradiance close to the water surface, above-water systems can directly derive remote sensing reflectance ($R_{rs}$) without complex atmospheric correction. Such systems are particularly suitable for optically complex and highly dynamic environments, such as rivers and estuaries. The development of the present GHRSS was inspired by above-water $R_{rs}$ measurements using field spectroradiometers such as the analytical spectral device (ASD) system [32]. In conventional ASD-based measurements, water-surface radiance, sky radiance, and downwelling irradiance are usually acquired through separate measurement procedures [33]. In this study, these three measurement processes were integrated into a single instrument platform to enable continuous and autonomous observation. Similar above-water radiometric systems have been developed previously, such as the open-source pySAS system [34], the Hyperspectral Surface Acquisition System (HyperSAS) [35] developed by Sea-Bird Scientific, and the three-channel RAMSES-SW system [36] developed by TriOS [37]. However, these systems are generally relatively expensive and may be less convenient for compact, fixed, high-frequency deployment in a sluice-controlled estuarine river environment. Therefore, the GHRSS proposed in this study was developed on the basis of established above-water reflectance measurement principles and previous community experience, with an emphasis on compact integration, autonomous operation, and high-frequency monitoring in a sluice-controlled river-estuary environment. Using paired GHRSS observations and in situ measurement data, we further established and validated DOC and POC retrieval models based on both empirical and machine learning approaches.

## 2. Ground-Based Hyperspectral Remote Sensing System Design and Development

### 2.1. Measurement Principle of the Above-Water Method

**Figure 1:**
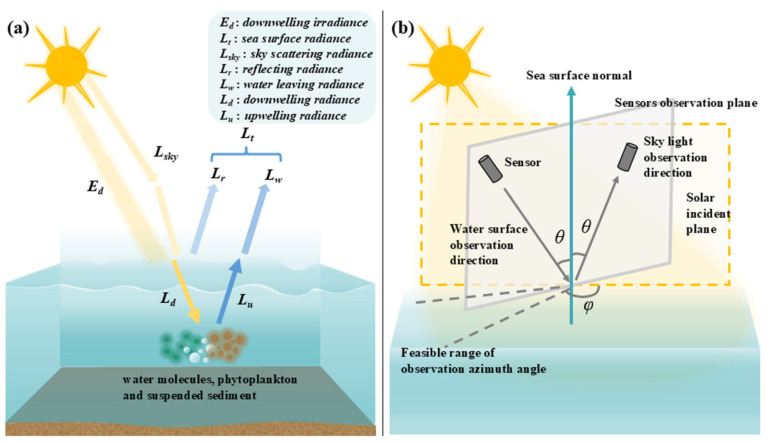
(**a**) Water surface signal schematic; (**b**) field observation geometry.

As shown in Figure 1a, solar downwelling radiation enters the water column and is backscattered by water molecules, phytoplankton, and suspended sediments. The portion that crosses the air–water interface and reaches the atmosphere is termed water-leaving radiance ($L_{w}$) [38]. Apparent optical properties such as $L_{w}$, normalized water-leaving radiance $\left(L_{wn}\right)$, and remote sensing reflectance ($R_{rs}$) are fundamental quantities in ocean-color remote sensing.

$R_{rs}$ is defined as Equation (1) [39]:

(1) $$R_{rs}\left(\lambda \right)=\frac{L_{w}\left(\lambda \right)}{E_{d}\left(\lambda \right)}$$

where $E_{d}\left(\lambda \right)$ is the total downwelling irradiance. Compared with $L_{w}$, $R_{rs}$ is less sensitive to variations in solar geometry and sky conditions. It also provides a consistent basis for comparing optical properties among different waters. For these reasons, $R_{rs}$ is widely used in field measurements and remote sensing applications. Accurate $R_{rs}$ requires reliable measurements of $L_{w}$ and $E_{d}$. Three main approaches are commonly used: in-water profiling method, skylight-blocking method, and above-water method [40].

The in-water profiling method measures the underwater light field at multiple depths and extrapolates it to the surface. It can also describe vertical variability in the light field. This method is relatively robust to environmental perturbations. However, it requires specialized instruments and complex deployment, and it is usually applied in waters deep enough for vertical profiling. In shallow or highly turbid waters, vertical gradients can be strong and extrapolation uncertainty may increase. The skylight-blocking method places a shading cone in front of the sensor. The sensor remains above the surface, while the cone tip is inserted into the water to measure $L_{w}$ more directly. This method reduces surface-reflected skylight and simplifies postprocessing. However, it is sensitive to surface waves and requires self-shading correction, which complicates accuracy assessment [41]. The above-water method is easy to operate and has low field cost. Therefore, it has been widely adopted [38,39,40,42], especially in optically complex coastal and estuarine waters where rapid deployment and repeated measurements are needed.

In above-water radiometry, the measured upwelling radiance above the surface includes three components: (i) the radiation contribution of direct solar reflection, (ii) the radiation contribution of skylight reflected by the sea surface, and (iii) the radiation contribution of water-leaving radiance. This can be expressed as Equation (2) [43,44]:

(2) $$L_{t}\left(\lambda \right)=L_{w}\left(\lambda \right)+\rho L_{sky}\left(\lambda \right)+L_{wc}\left(\lambda \right)+L_{g}\left(\lambda \right)$$

where $L_{t}$ denotes the total upwelling radiance above the water surface. $\rho L_{sky}$ is the signal that enters the sensor after skylight is reflected by the water surface, containing no information about the water body, wherein ρ represents the skylight reflectance at the air–water surface. Typical ρ values are about 0.022 under calm conditions, about 0.025 for wind speed below 5 m s^−1^, and about 0.026–0.028 for wind speed around 10 m s^−1^ [45]. $L_{wc}$ represents the whitecap and is often negligible under low-wind conditions. $L_{g}$ represents sun glint, which can be reduced by controlling the viewing geometry. Following the SeaWiFS Ocean Optics Protocols (Figure 1b), the relative azimuth between the viewing plane and the solar principal plane is set to 135°, and the viewing angle is set to 40° from nadir [46,47]. This geometry avoids most sun glint and minimizes $L_{g}$. Under these conditions, Equation (2) can be simplified to:

(3) $$R_{rs}\left(\lambda \right)=\frac{L_{t}\left(\lambda \right)-\rho L_{sky}\left(\lambda \right)}{E_{d}\left(\lambda \right)}$$

Equation (3) indicates that accurate $R_{rs}\left(\lambda \right)$ depends on reliable measurements of $L_{t}\left(\lambda \right)$, $L_{sky}\left(\lambda \right)$, and $E_{d}\left(\lambda \right)$, as well as an appropriate estimate of ρ. The observation geometry and skylight-reflection correction adopted here are generally consistent with commonly used recommendations in previous above-water radiometry studies [38,39,40]. Nevertheless, uncertainties may still remain due to variations in sky conditions, surface roughness, empirical assumptions in ρ, and residual sun glint effects under non-ideal field conditions [38,40,42].

### 2.2. Overall Design

**Figure 2:**
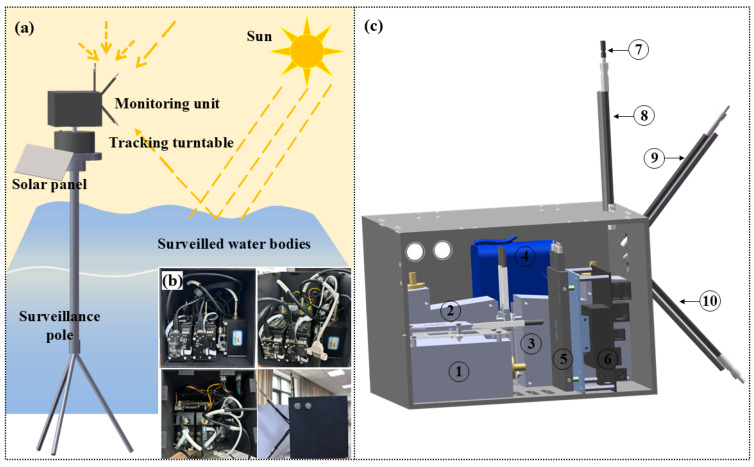
(**a**) Schematic diagram of the system; (**b**) photo of the monitoring unit; (**c**) 3D model of the monitoring unit. The monitoring unit is composed of the following components internally: 1: water spectrometer; 2: sky spectrometer; 3: irradiance spectrometer; 4: battery; 5: hard drive; 6: microcontrol board; 7: cosine corrector; 8, 9 and 10: optical fibers.

The GHRSS was designed in accordance with the principle of the above-water method, its framework and working process are illustrated in Figure 2a. It includes a surveillance pole, a solar panel, a tracking turntable, and a monitoring unit. Figure 2b is a physical image of the monitoring unit, and Figure 2c is a three-dimensional model of the monitoring unit, which shows the internal electronic and communication circuits of the monitor system, as well as the microspectrometer and power supply. The monitoring unit is the core of the entire observation system, and it mainly consists of four modules: (i) a data acquisition module, (ii) a control module, (iii) a power supply module, and (iv) a communication module. In addition, these instruments are housed in a black nylon instrument box, with the monitoring unit having an overall size of approximately 19 cm × 19 cm × 18.2 cm and a total weight of about 2.3 kg.

The data acquisition module is designed to derive $R_{rs}$, including three optical channels: water surface radiance sensor, skylight radiance sensor, downwelling irradiance sensor. These channels are connected to the spectrometers through three matched optical fibers, and a cosine corrector is mounted on the irradiance channel. All three sensors are miniature fiber-optic spectrometers with a crossed Czerny–Turner (CT) optical configuration. Each spectrometer weighs approximately 0.7 kg. The spectral detection range covers the visible and near-infrared wavelengths, with a maximum spectral resolution of up to 0.4 nm. The integration time can be set as low as 10 μs, and the fiber input interface is an SM905 connector. To obtain the radiometric quantities required for $R_{rs}$ calculation, including $L_{t}$, $L_{sky}$, and $E_{d}$, the fiber probe of the water-surface radiance sensor is set at a nadir angle of 40° to measure the total upwelling signal from the water surface, and the fiber probe of the sky radiance sensor is maintained at a zenith angle of 40° to measure scattered skylight. For the downwelling irradiance measurement, a cosine corrector is attached to the fiber probe, which is oriented vertically upward to collect radiance uniformly over the upper hemispherical (180°) field of view.

The operation of the data acquisition module is mainly operated by a microcontrol board equipped with a Windows 10 system and an Intel i7 processor, with a maximum full-load power consumption of approximately 40 W. Since the spectrometers can operate through standard USB drivers, they can be easily integrated with the controller board. We installed self-developed software on the board to operate the sensors.

The communication module is used to enable wireless data transfer to the cloud server, it includes a Quectel EC800M 4G unit with a 4G nano-SIM IoT card. Via 4G signals, the collected data can be routed to the terminal computer in real time through the TCP/IP protocol and simultaneously backed up to the cloud platform through the MQTT protocol, enabling remote monitoring, cloud backup, and network connectivity.

Considering that the system needs to be deployed in the field for long-term measurements, the issue of power supply endurance must be addressed. Here, a battery equipped inside the system and a solar panel are used to ensure reliable operation under different weather conditions. When sunlight is sufficient, the electrical energy from the solar panel is stored in the 12 V battery for use when sunlight is insufficient. In practical field applications, the internal battery alone can support continuous system operation for approximately 14 h, which is equivalent to about 2 days under the present observation schedule.

When the battery capacity drops below 10%, the circuit between the solar panel and the battery will connect, allowing the battery to collect and store energy; conversely, if the battery capacity exceeds 95%, the circuit between the solar panel and the battery will automatically disconnect to prevent overcharging and potential damage to the battery.

Sun glint is difficult to measure and quantify accurately. In the above-water method, its influence is therefore reduced by adjusting the observation geometry. The fiber-probe orientations described above already ensure a viewing angle of 40° relative to the sea-surface normal. In addition, a tracking turntable is required to maintain a relative azimuth angle of 135° between the viewing plane of sensors and the solar principal plane. After acquiring positioning information from a GPS module, the theoretical solar position is calculated from the geographic coordinates and measurement time using standard astronomical equations. This calculation provides the solar elevation and azimuth angles, which are then used to determine the required rotation angle of the platform [48]. The tracking turntable is driven by a stepper motor, a reducer, and a motor driver, allowing the sensor viewing plane to be adjusted automatically to meet the prescribed observation geometry.

In summary, the above components and functionalities form an integrated system that allows the monitoring unit to capture spectral information of the target water body. The main optical and technical specifications of the GHRSS are summarized in Table 1. Overall, the design of the system achieves three goals: low cost, low energy and low footprint.

### 2.3. Data Acquisition Software

**Figure 3:**
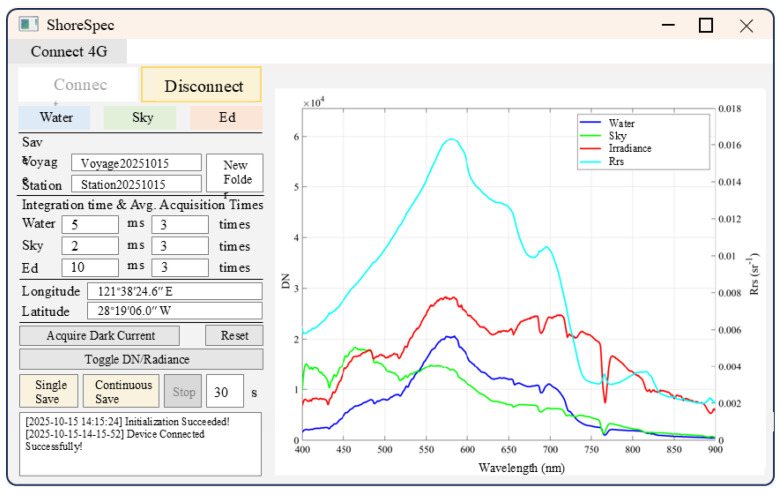
Acquisition software UI.

To enable effective acquisition of water-surface remote sensing reflectance, dedicated data acquisition software was developed using Visual C++. The overall interface of the software is shown in Figure 3. The software consists of three main functional components. The first component is system control of the GHRSS observation platform. Through the software interface, the three spectrometers can be activated, and key acquisition parameters can be configured, including integration time, number of averaged measurements, and time interval for continuous data storage. The second component provides real-time preprocessing, visualization, and storage of the acquired spectral data. The third component enables wireless data transmission using 4G and GPRS modules. Based on the TCP/IP protocol, the software can transmit data to a remote server and receive control commands from the server.

### 2.4. Radiometric Calibration

All optical channels record raw digital numbers (DNs). Prior to the calculation of $R_{rs}$, radiometric calibration was performed to convert DNs into radiance and irradiance [49]. The radiance and irradiance sensors were calibrated independently using a standard lamp and a diffuse reflectance reference panel.

**Figure 4:**
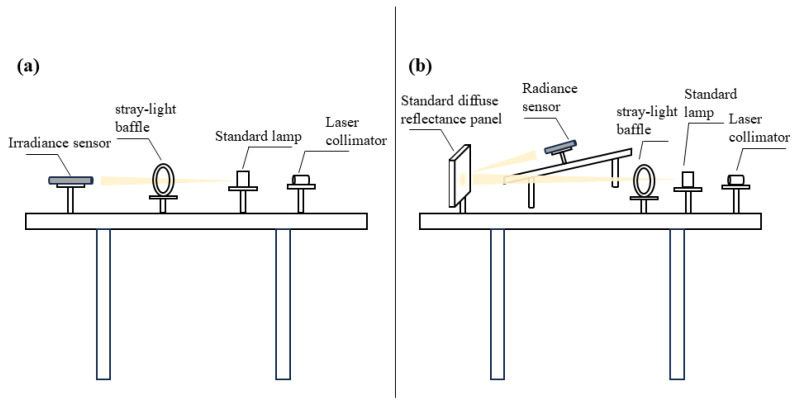
Schematic diagram of radiometric calibration. (**a**) Irradiance sensor; (**b**) Radiance sensor.

As shown in Figure 4, the irradiance sensor was calibrated by placing the sensor, a stray-light baffle, and the standard lamp on an optical rail, with the fiber probe aligned with the lamp filament. The radiance sensor was calibrated using a standard lamp and a diffuse reflectance panel. The lamp illuminated the panel vertically, and the radiance sensor measured the reflected radiance at a viewing angle of 45°. The responsivities of the irradiance and radiance sensors were calculated using Equations (4) and (5), respectively.

(4) $$R_{E}\left(\lambda \right)=\frac{E_{0}\left(\lambda \right)}{N_{E0}\left(\lambda \right)}\frac{d_{0}^{2}}{d_{1}^{2}}$$

(5) $$R_{L}\left(\lambda \right)=\frac{E_{0}\left(\lambda \right)}{N_{L0}\left(\lambda \right)}\rho_{panel}\left(\lambda \right)\frac{d_{0}^{2}}{\pi d_{2}^{2}}$$

where $E_{0}$ is the calibrated irradiance of the standard lamp at the reference distance $d_{0}=50\;\mathrm{cm}$; $N_{E0}$ and $N_{L0}$ are the signals measured by the irradiance and radiance sensors, respectively; and $\rho_{panel}$ is the reflectance factor of the diffuse reference panel traceable to a national metrology institute.

**Figure 5:**
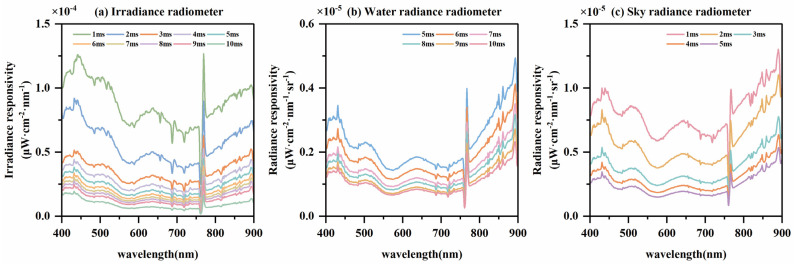
Spectral responsivity curves of the radiance and irradiance radiometer.

The calibration lamp was a 1000 W quartz–tungsten–halogen lamp traceable to the National Institute of Metrology of China, with a calibrated spectral range of 250–2500 nm. The diffuse reference panel had a nominal reflectance of 99% and dimensions of 400 mm × 400 mm. The resulting spectral responsivities of the radiance and irradiance sensors are shown in Figure 5. It should be noted that the three optical channels were radiometrically calibrated prior to experimental application. Because the observation period analyzed in this study was relatively limited, no additional long-term sensor-drift correction procedure was implemented in the current preprocessing workflow. Nevertheless, differential drift among the three sensors may remain a source of uncertainty during extended deployment, and future work should include regular recalibration and cross-sensor consistency checks to better control this issue.

## 3. Experimental Application for Monitoring DOC and POC

### 3.1. Experiment Area

**Figure 6:**
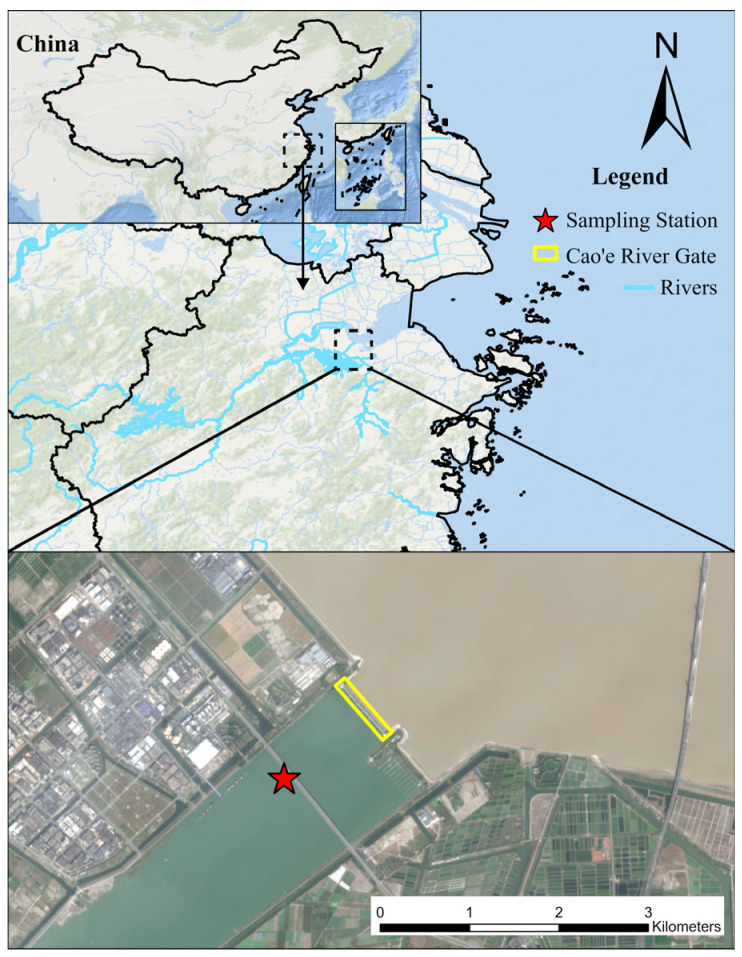
Experiment area and the location of sampling point.

To evaluate the performance of the GHRSS for water environment monitoring, a field experiment was conducted in the water area upstream of the Cao’e River sluice ($120{}^{\circ}44^{\prime }\;E,\;30{}^{\circ}13^{\prime }\;N$), China (Figure 6). The GHRSS was deployed at this site to carry out continuous observations.

The Cao’e River is a major river discharging into Hangzhou Bay and the Zhejiang coastal waters. Its upper reaches are characterized by mountainous stream conditions, while the lower reaches form a tidal river section. The main stem of the Cao’e River is approximately 197 km long, with a drainage area of about 6080 km^2^, and it ultimately enters the Qiantang River estuary through the Cao’e River sluice [50]. As an important river system that supports both ecological functions and human activities, water quality in the Cao’e River is jointly influenced by river discharge, tidal mixing, and anthropogenic inputs, including agricultural, industrial, and urban sources [51]. The Cao’e River sluice project is located at the river mouth and serves multiple purposes, including flood control, waterlogging prevention, water resource regulation, environmental improvement, and navigation [52]. The presence of the sluice increases water residence time upstream. During the rainy season or under strong tidal forcing, gate operations such as sluice opening for discharge can induce rapid changes in hydrodynamic conditions and water quality. As a result, the hydrological and water quality conditions in the study area are jointly driven by tidal fluctuations and sluice regulation, which may lead to pronounced short-term variability.

Overall, the highly dynamic nature of this sluice-controlled estuarine river reach provides an ideal test site for evaluating the capability of the GHRSS to capture real-time and high-frequency variations in DOC and POC.

### 3.2. Optical Data and Water Sampling Collection

In order to establish inversion models for the concentration of DOC and POC using reflectance spectra of water bodies for tracking their variations, it is necessary to acquire $R_{rs}$ of the water surface and the corresponding in situ data.

#### 3.2.1. Optical Data Acquisition and Quality Control

Relying on the GHRSS, spectrum data under complex weather conditions were continuously and stably collected by a high-resolution spectrometer placed on the bridge in front of the Cao’e River sluice, approximately 30 m above the water surface. In this study, the spectrometer continuously obtained $R_{rs}\left(\lambda \right)$ at 3 min intervals. The GHRSS datasets were acquired from 9:00 to 16:00 to avoid sensor deviation and low sunlight. Given the near-water observation setup, atmospheric path effects are reduced compared with higher-altitude observations. No obvious droplet accumulation on the optical interfaces was observed during the valid measurement periods used in this study, although moisture condensation or aerosol deposition may remain a potential source of uncertainty during long-term deployment. This reduces the need for the complex preprocessing steps commonly required in satellite or higher-altitude water remote sensing, particularly atmospheric correction [53,54,55], and thus enables the instrument to capture stronger water-leaving radiance signals. Consequently, it becomes easier and more accurate to develop retrieval models for DOC and POC parameters in riverine waters discharging into the sea.

**Figure 7:**
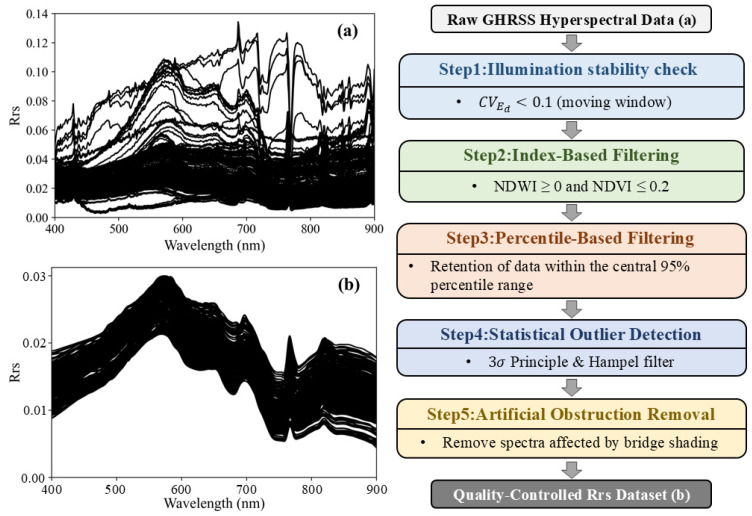
Procedure of data quality control. (**a**) Continuously measured Rrs before processing. (**b**) Quality-controlled Rrs result.

A comprehensive quality-control (QC) procedure was applied to the GHRSS dataset to ensure the reliability of the high-frequency hyperspectral $R_{rs}\left(\lambda \right)$ measurements. The QC strategy was designed to remove data affected by unstable illumination, extreme values, building obstructions, and non-water signals, while preserving the natural temporal variability of the observations. (1) Measurements acquired under unstable illumination conditions were screened based on the temporal stability of downwelling irradiance ($E_{d}$). For each observation, a short moving window was constructed, and the coefficient of variation (CV) of $E_{d}$ within the window was calculated [56]. Records associated with rapid irradiance fluctuations, mainly caused by passing clouds, were identified and removed. (2) Index-based filtering was applied using the normalized difference water index (NDWI) and the normalized difference vegetation index (NDVI) to remove spectral data affected by non-water targets and interference from surface phytoplankton. (3) A percentile-based filter was employed to remove extreme values, retaining only measurements within the central 95% percentile range of the selected spectral features. (4) Statistical outlier detection was performed on the high-frequency time series using a combination of a sigma-based criterion and the Hampel filter [57]. This approach effectively removed isolated abnormal points while maintaining the overall temporal structure of the dataset. (5) Measurements affected by intermittent shading from the bridge structure were identified and removed based on synchronous abrupt decreases in downwelling irradiance and upwelling radiance, which are characteristic of obstruction events [56,58]. After applying these QC steps, a consistent and high-quality hyperspectral dataset was obtained for subsequent DOC and POC retrieval. The overall QC workflow is illustrated in Figure 7.

The quality-controlled $R_{rs}\left(\lambda \right)$ spectra exhibit a typical estuarine spectral shape, characterized by low reflectance in the blue region, a pronounced peak in the green wavelengths, and non-negligible near-infrared reflectance associated with high suspended particulate matter concentrations. A narrow spectral artifact occasionally observed around 760 nm is attributed to instrument-related effects. To avoid its potential influence on subsequent analyses, spectral bands in this wavelength region were excluded from DOC and POC retrieval. Overall, the spectral features are consistent with those reported for highly turbid river-estuary systems [59,60,61].

#### 3.2.2. Water Sampling and DOC/POC Sample Collection

The in situ data were collected at Cao’e River every 30 min during the observation periods 17 October 2025 and 15 to 20 November 2025. Surface water samples were collected using a 2.5 L plastic water collector. DOC and POC samples were filtered through Whatman GF/F glass fiber filters (nominal pore size: 0.7 μm) with diameters of 47 mm and 25 mm, respectively. For DOC preservation, the filtrates were transferred to brown glass vials, whereas for POC, the particulate matter retained on the filters was wrapped in aluminum foil. All DOC and POC samples were subsequently stored frozen at −20 °C until analysis. Prior to use, all filters were preburned in a muffle furnace at 450 °C for 5 h to remove residual organic contaminants [62]. DOC concentrations were determined by high-temperature catalytic oxidation (HTCO) using a TOC-Analyzer [63,64]. For POC, samples were first acidified to remove particulate inorganic carbon (PIC), followed by drying. Subsequently, the carbon content (expressed as a percentage) of the POC samples was measured using the combustion method with an elemental analyzer [65,66]. Finally, a total of 90 paired DOC and POC samples were obtained from the observation periods on 17 October 2025 and 15–20 November 2025.

### 3.3. Model Development and Validation

Currently, there are two major methods for estimating DOC and POC, namely the empirical method and the machine learning method [67,68,69]. Generally, developing an empirical model requires two steps [54,70]: (1) selecting the most sensitive spectral index to the measured DOC and POC from a series of spectral indices, including signal band, band ratio, sum or difference of two bands, and normalized difference index; (2) determining the relationships between the optimal spectral indices and the measured DOC and POC using linear and non-linear fitting models, including linear, quadratic, logarithmic, exponential, and power functions, and selecting the best model for each parameter based on the highest R^2^ and the lowest error. Considering that DOC and POC are non-optically active, the machine learning method may enhance the reliability and accuracy of inversion results [71].

The $R_{rs}$ data were temporally matched with the measured DOC and POC concentrations, resulting in 90 paired samples for inversion model development. These samples were randomly divided into a training set (*N* = 63) and an independent test set (*N* = 27). The training set was used for model calibration and parameter optimization, while the independent test set was reserved exclusively for final model evaluation and was not involved in model training, cross-validation, or parameter tuning. Specifically, five-fold cross-validation was performed only within the training set to identify the optimal hyperparameters. Once the optimal parameters were obtained, the model was retrained on the entire training set and subsequently applied to the independent test set to generate predictions and evaluate the final model performance. This strategy was adopted to evaluate model performance within the current dataset.

**Figure 8:**
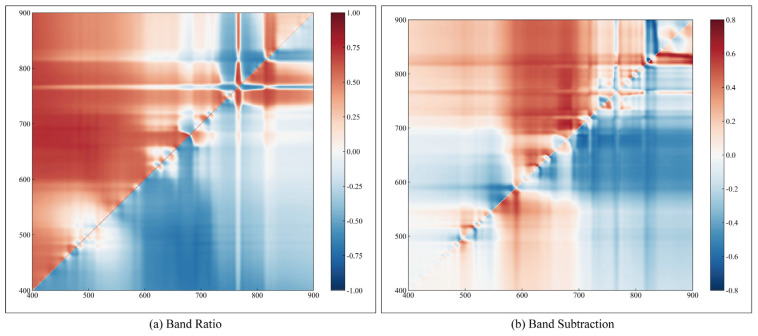
Correlation analysis of the best band transformations for DOC and POC. (**a**) DOC; (**b**) POC.

To identify spectrally sensitive variables for subsequent model development, correlation analysis was conducted between DOC and POC concentrations and the $R_{rs}$ spectra measured by the GHRSS. Four spectral indices, including single band, sum or difference of two bands, and ratio of two bands, were used to fit in situ DOC and POC. The best-performing spectral indices identified from this analysis are summarized in Table 2. Among the four types of spectral indices examined, the band-ratio index showed the strongest correlation with DOC, whereas the band-difference index showed the strongest correlation with POC. For visualization, these correlation results are presented in Figure 8.

**Figure 9:**
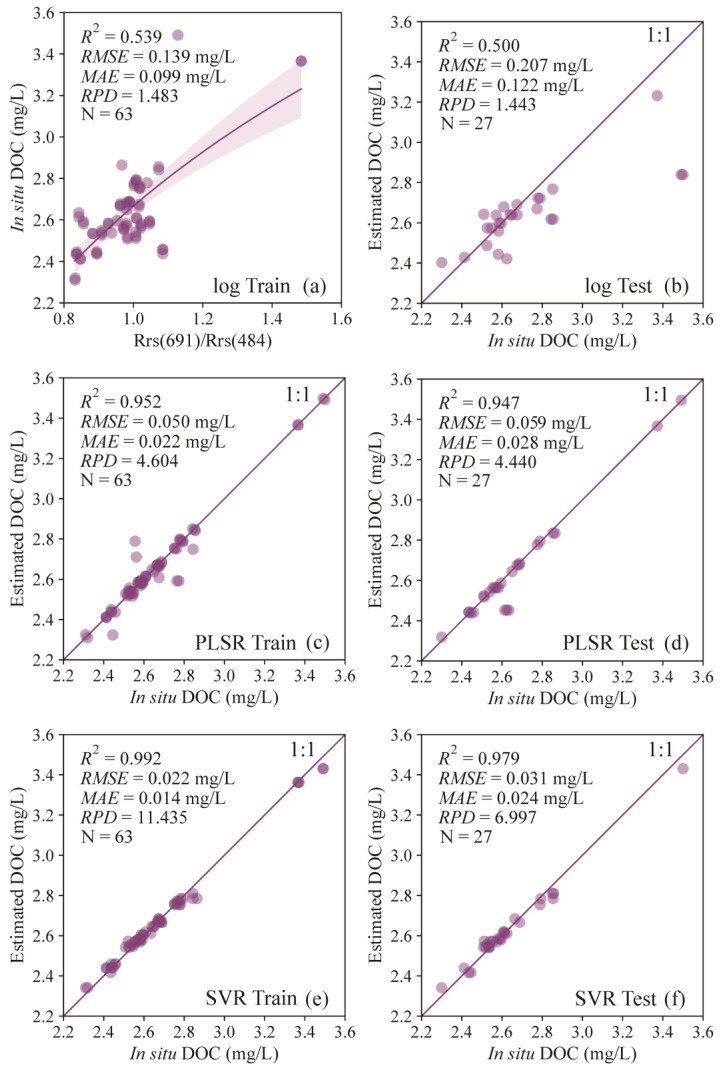
Scatterplots of development (*N* = 63) and test (*N* = 27) based on DOC logarithmic model (**a**,**b**), PLSR model (**c**,**d**) and the SVR model (**e**,**f**).

**Figure 10:**
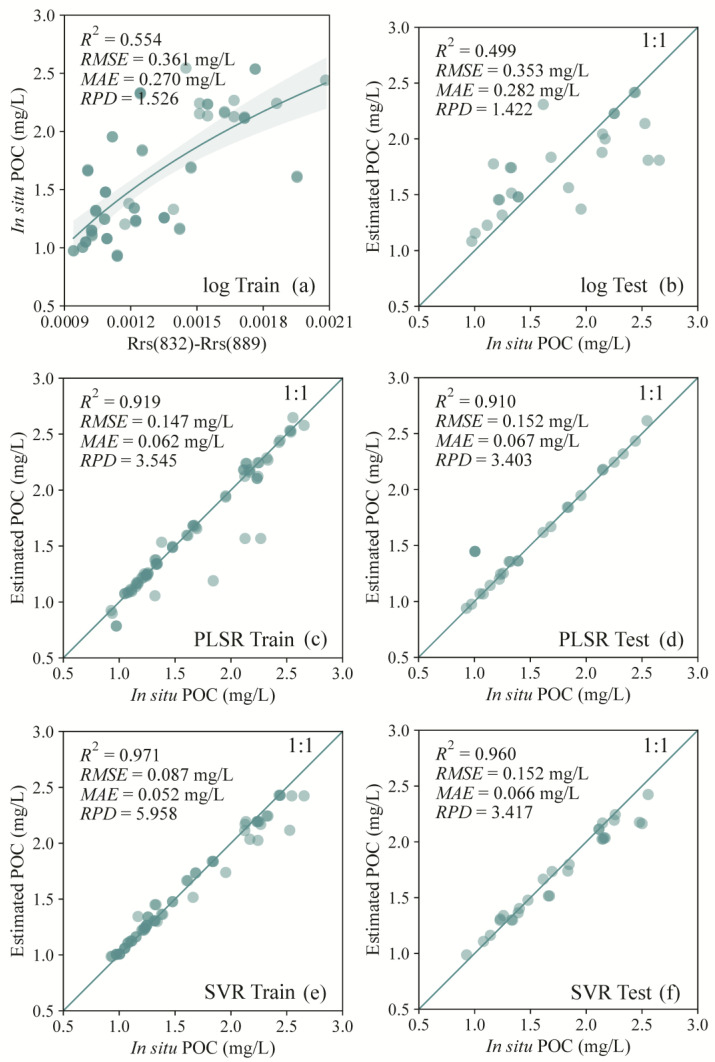
Scatterplots of development (*N* = 63) and test (*N* = 27) based on POC logarithmic model (**a**,**b**), PLSR model (**c**,**d**) and the SVR model (**e**,**f**).

The optimal spectral indices for DOC and POC were used to construct empirical statistical models, including linear, logarithmic, exponential, and power models. Among these models, the logarithmic model exhibited the best performance for both DOC and POC. The specific model formulations are presented as follows:

(6) $$C_{DOC}=1.432\ln[\frac{R_{rs}\left(691\right)}{R_{rs}\left(484\right)}]+2.666$$

(7) $$C_{POC}=1.678\ln\left[R_{rs}\left(832\right)-R_{rs}\left(889\right)\right]+12.77$$

where $C_{DOC}$ and $C_{POC}$ denote the concentrations of DOC and POC, respectively. For DOC, the best empirical model was a logarithmic function (Equation (6)) using the band ratio of 691 nm and 484 nm, with an R^2^ of 0.539, an RMSE of 0.139 mg/L, and an MAE of 0.099 mg/L (Figure 9a). For POC, the best model was also a logarithmic function (Equation (7)), constructed using the band difference between 832 and 889 nm, with an R^2^ of 0.554, an RMSE of 0.361 mg/L, and an MAE of 0.270 mg/L (Figure 10a). Although the logarithmic model outperformed the other empirical formulations, the overall retrieval performance of the empirical approaches remained limited for both DOC and POC.

Therefore, partial least squares regression (PLSR) and support vector regression (SVR) were further introduced to improve model accuracy. These two methods were selected because they are well suited to the characteristics of the present dataset. PLSR is a representative linear multivariate method that can effectively handle high-dimensional and strongly collinear hyperspectral variables while reducing dimensionality during model construction, which makes it particularly suitable for relatively small sample spectral studies. In contrast, SVR is capable of modeling indirect and partly non-linear relationships between spectral information and water quality variables, and it generally performs robustly under limited sample conditions. Because DOC and POC are not optically active constituents and their relationships with reflectance are mainly indirect, the combined use of PLSR and SVR allowed us to compare one physically interpretable linear method with one robust non-linear method under the same dataset. Considering the sample size (*N* = 90) and the objective of providing a focused yet representative methodological comparison, we retained these two methods for detailed analysis rather than expanding to a larger set of algorithms.

For model implementation, the full-spectrum remote sensing reflectance data were directly used as input variables for PLSR, because this method inherently combines dimensionality reduction with regression. For SVR modeling, the input variables were further selected from the correlation analysis results summarized in Table 2, with consideration of spectral robustness and physical interpretability. Considering the spectral resolution limitations of the miniature spectrometers, immediately adjacent band combinations were not retained in the revised SVR model in order to reduce the risk of fitting instrumental noise. Accordingly, the revised SVR model for DOC was constructed using $R_{rs}$(604), $R_{rs}$(691)/$R_{rs}$(484), and $R_{rs}$(592) + $R_{rs}$(705), whereas the revised SVR model for POC was constructed using $R_{rs}$(819) and $R_{rs}$(832) − $R_{rs}$(889). Compared with the empirical models, both PLSR and SVR achieved substantially improved retrieval performance, with R^2^ values exceeding 0.9 for both DOC and POC. Among these models, the SVR algorithm showed the best performance for DOC retrieval, achieving the highest R^2^ (0.992) and RPD (11.435), as well as the lowest RMSE (0.022 mg/L) and MAE (0.014 mg/L) (Figure 9e). Likewise, SVR also produced the best retrieval results for POC, yielding an R^2^ of 0.971, an RMSE of 0.087 mg/L, an MAE of 0.052 mg/L, and an RPD of 5.958 (Figure 10e).

To validate the accuracy and robustness of the DOC and POC estimation models, the logarithmic, PLSR, and SVR models were evaluated on the independent test set using R^2^, RMSE, MAE, and RPD (Table 3), as shown in Appendix A. This test set was reserved exclusively for final evaluation and was not involved in model training, cross-validation, or parameter tuning. For DOC, the SVR model exhibited higher accuracy (R^2^ = 0.979, RMSE = 0.031 mg/L, MAE = 0.024 mg/L, and RPD = 6.997) than the logarithmic model (R^2^ = 0.500, RMSE = 0.207 mg/L, MAE = 0.122 mg/L, and RPD = 1.443) and PLSR model (R^2^ = 0.947, RMSE = 0.059 mg/L, MAE = 0.028 mg/L, and RPD = 4.440) (Figure 9b,d,f). In addition, compared with the logarithmic and PLSR models, the DOC values retrieved by the SVR model were more evenly distributed around the 1:1 line, indicating better agreement with the in situ DOC measurements. For POC, the SVR model likewise achieved the best overall estimation performance, with the highest $R^{2}$(0.960) and RPD (3.417), and the lowest MAE (0.066 mg/L). Its RMSE (0.152 mg/L) was identical to that of the PLSR model but markedly lower than that of the logarithmic model (0.353 mg/L) (Figure 10b,d,f). Moreover, compared with the logarithmic and PLSR models, the POC values estimated by the SVR model were more closely distributed around the 1:1 line, further demonstrating its superior predictive capability and robustness.

Therefore, to minimize estimation error, the SVR model exhibiting the highest determination coefficient and lowest errors was selected for quantifying the dynamics of DOC and POC. This model demonstrated satisfactory performance and strong applicability.

### 3.4. Minute-Scale Time Series of DOC and POC Variation

**Figure 11:**
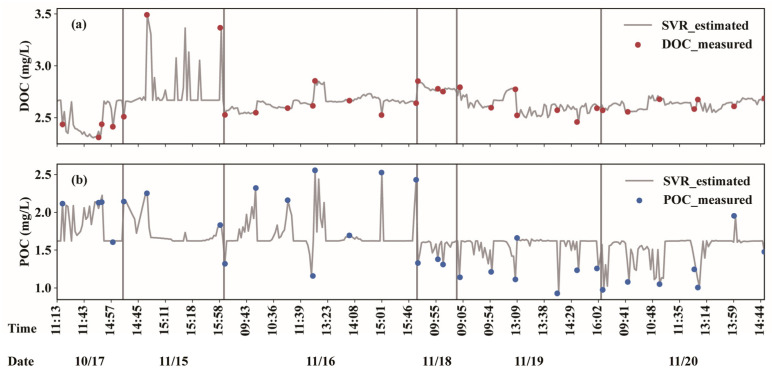
Minute-scale time series of DOC and POC derived from the GHRSS and SVR estimation model. (**a**) DOC; (**b**) POC.

The minute-scale time series variation of DOC and POC for the Cao’e River was produced from the GHRSS data using the developed SVR models. Different short-term dynamic characteristics of DOC and POC in the Cao’e River Gate are shown in Figure 11 for the observation period.

Overall, the dynamic changes in the predicted values from the SVR algorithm are largely consistent with the actual measured values. Both DOC and POC exhibited pronounced sub-daily variability, with clear fluctuations within each daily observation window. DOC varied within a relatively narrow range of 2.31–3.49 mg/L. In contrast, POC displayed larger short-term variability, ranging from 0.92–2.55 mg/L, with more frequent and sharper changes on timescales of 1 h. Several episodic peaks and troughs were observed (Figure 11b), indicating that particulate carbon dynamics in the sluice-controlled reach can respond rapidly to changing hydrodynamic conditions.

DOC and POC also showed different temporal behaviors. On multiple days, changes in POC were more abrupt than those in DOC, suggesting stronger sensitivity of particulate carbon to short-term forcing such as resuspension and mixing, whereas DOC tended to vary more smoothly. These high-frequency records demonstrate that substantial DOC/POC variability occurs within hours, which would likely be missed by conventional low-frequency sampling.

## 4. Discussion

### 4.1. Consistency Between In Situ ASD and GHRSS

To evaluate the accuracy of the above-water reflectance measured by the GHRSS, we compared the data obtained by the surface object spectrometer ASD (FieldSpec 4 Hi-Res spectroradiometer, Analytical Spectra Devices, Inc., Malvern, UK) and the GHRSS at the same sampling site. Both instruments followed the same observation period and were operated under comparable illumination conditions. The comparison provides a direct assessment of the radiometric performance of the GHRSS, including its irradiance-related stability and the overall reliability of retrieval in field conditions.

**Figure 12:**
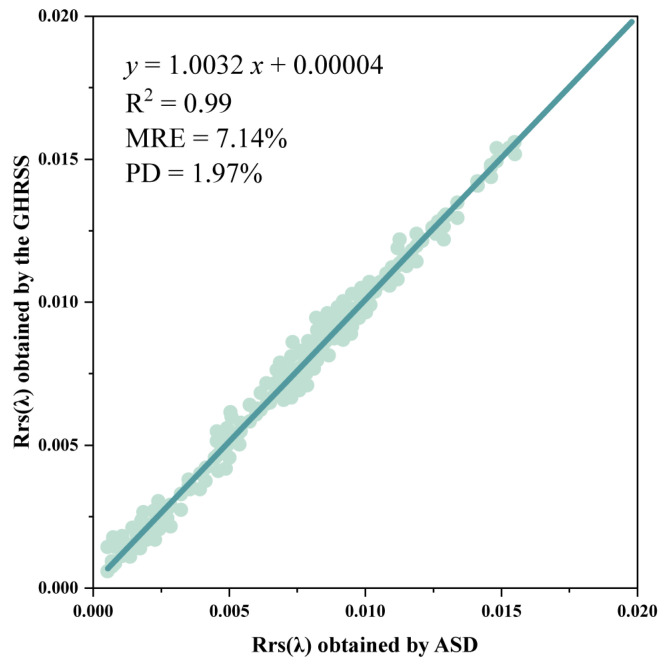
Comparison of the *R_rs_*(λ) obtained by ASD and the GHRSS.

The results showed satisfactory consistency (Figure 12). The fitted regression line is *y* = 1.0032 *x* + 0.00004 with R^2^ = 0.99, indicating that the GHRSS-derived $R_{rs}$ closely matches the ASD-derived *R_rs_* over the full reflectance range. The slope is very close to 1, suggesting that the scaling of *R_rs_* is consistent between instruments and that the radiometric calibration and irradiance normalization of the GHRSS are reliable. In addition, the small intercept (~4 × 10^−5^) implies only a minor systematic offset. Error metrics further support the consistency, with a mean relative error (MRE) of 7.14% and a percent difference (PD) of 1.97%, which are within an acceptable range for above-water hyperspectral observations in optically complex waters.

The remaining discrepancies likely arise from practical field factors rather than fundamental system bias. First, small differences in observation geometry and viewing footprint can lead to slight variations, especially in turbid and spatially heterogeneous waters. Second, rapid short-term changes in illumination may introduce residual mismatches even under nominally synchronous measurements. Third, instrument-specific characteristics (e.g., fore-optics alignment, stray light, and sensor noise) can also contribute, particularly at low *R_rs_* levels where relative errors tend to increase. Overall, the high consistency between ASD and GHRSS supports the use of the GHRSS for continuous, high-frequency *R_rs_* monitoring and provides a solid foundation for subsequent DOC and POC retrieval in the study area.

### 4.2. Uncertainty of High-Frequency POC Fluctuations

The high-frequency time series derived from the GHRSS suggest pronounced short-term variability in both DOC and POC, with POC showing more abrupt fluctuations than DOC. Such variability may reflect the rapid response of particulate matter to changing hydrodynamic conditions in the sluice-controlled reach. In particular, some of the abrupt increases in POC may be related to gate-opening-induced water stirring or sediment resuspension, both of which can rapidly modify the concentration and optical influence of suspended particulate matter.

At the same time, these short-term fluctuations should be interpreted with caution. The current dataset does not allow the observed changes to be attributed unambiguously to environmental processes alone. In addition to actual hydrodynamic variability, uneven illumination conditions, spatial heterogeneity of the water body, and other local observational artifacts may also contribute to the observed signals. Moreover, because the observation site is located near a sluice structure, potential interference from artificial light or local scene complexity cannot be completely excluded. Therefore, although the high-frequency observations may capture real particulate dynamics, the present results should be regarded as a preliminary indication rather than definitive proof of the underlying mechanisms.

Future work should combine the optical observations with gate-operation records, synchronous hydrodynamic measurements, and additional in situ particulate observations to better distinguish real environmental fluctuations from potential observational artifacts.

### 4.3. Advantages and Limitations of the Proposed GHRSS

Inland-water monitoring of organic carbon is important for understanding aquatic carbon cycling and supporting water environment management. However, time-consuming and spatiotemporally limited manual sampling methods, weather-vulnerable satellite and UAV remote sensing methods, and underwater sensors being limited by adhesive fouling have resulted in the requirements for rapid and reliable DOC and POC monitoring not being met. In general, the GHRSS has several major advantages as follows.

First, the GHRSS provides an efficient and easy-operation tool for rapid observation of organic carbon content under normal temperature and pressure, which can instantaneously and continuously provide high-frequency hyperspectral data in an environmentally friendly manner and at low cost. Traditional laboratory analysis requires labor-intensive and time-consuming field sampling and filtration. For POC in particular, samples usually undergo multiple pretreatment steps, such as acid fumigation, rinsing, and drying. In addition, consumables such as filters and sample bottles often need to be precleaned (e.g., acid-washed and/or precombusted) to reduce contamination. These procedures increase labor demand, reduce monitoring efficiency, and may still introduce contamination risks. Similarly, retrieving DOC and POC from satellite or UAV images also typically requires a series of complex preprocessing steps, including radiometric calibration, atmospheric correction, and geometric correction [53,54]. In contrast, the proposed GHRSS can directly provide above-water hyperspectral measurements under ambient conditions simply by setting the measurement frequency. This avoids sample handling and associated contamination risks, and it substantially improves monitoring efficiency for high-frequency observations. Moreover, the maintenance of the GHRSS is less expensive than those of underwater automatic instruments. This feature makes the GHRSS more economical and automatic.

Second, using the GHRSS for high-frequency and continuous observation was conducive to tracking complete variation and validating and supplementing the dataset collected by traditional monitoring methods. Compared with discrete sampling, the GHRSS can capture short-lived fluctuations and episodic events that are easily missed at low sampling frequencies. In addition, satellite remote sensing often provides only a limited number of valid observations within a year because of cloud contamination and revisit intervals [53]. Therefore, GHRSS time series can serve as an important supplement to satellite products by filling temporal gaps and providing dense matchups for calibration and validation.

In addition, the GHRSS with proximal and non-contact sensing improved the accuracy and reliability of DOC and POC estimation. Only a small fraction (less than 10%) of the top-of-atmosphere signal over water is contributed by the water-leaving component. Therefore, retrieval accuracy can be strongly affected by atmospheric correction errors. Although the ASD water-spectra measurements and underwater sensors were not limited by the atmosphere, ASD measurements require manual adjustment of the viewing geometry to avoid sun glint. This is inconvenient in routine monitoring and may lead to slight inconsistencies in the viewing direction and sampling footprint among measurements, which can introduce additional uncertainty and bias. The application of the underwater sensor was limited by its cost, and it can be vulnerable to hydrodynamic disturbance and often suffer from biofouling-related degradation in data quality. Fortunately, the observation height of the GHRSS not only reduced the influence of atmospheric aerosols, increased the signal-to-noise ratio of the water body, and avoided wind-wave erosion and biofouling-related degradation but also automatically adjusts the viewing geometry to avoid sun glint, which improved the accuracy and efficiency of spectrum data.

There are still some limitations in the precision and application of the GHRSS. First, the current deployment is based on point measurements, which limits spatial representativeness. Future work should expand the observation strategy from a single point to spatially distributed measurements, for example, by deploying multiple stations or using a scanning configuration. Second, a larger and more diverse set of water samples covering optically complex conditions is required to improve DOC and POC retrieval. The representativeness and diversity of the training dataset directly affect the stability, robustness, and transferability of proximal sensing models. In addition, the paired samples used in this study were collected continuously at 30 min intervals during a limited observation period at a single study site. Under such conditions, temporal autocorrelation is likely present in the dataset, and a purely random split between the training and test sets may lead to an overly optimistic estimate of model performance because temporally adjacent samples can share similar hydro-optical conditions. Therefore, the reported model accuracy should be interpreted primarily as reflecting predictive performance within the same study area and observation period, rather than full generalization capability to unseen water bodies. Moreover, the present DOC and POC retrieval models should be regarded primarily as site-specific relationships for the upstream water body of the Cao’e River Gate. Because the optical and biogeochemical conditions may differ substantially on the other side of the gate, direct application of the current algorithm to that water body should be treated with caution. More generally, although the GHRSS hardware framework can be deployed at other sites, the retrieval algorithm itself is not necessarily directly transferable to optically or hydrodynamically different environments. Application at a different site would require new in situ DOC/POC sampling and synchronous spectral observations for site-specific calibration or, at minimum, independent validation. Future work should incorporate temporally constrained validation strategies and broader multisite datasets to provide a more rigorous assessment of model robustness and transferability. Third, the retrieval algorithms could be integrated into the GHRSS software v 1.0 to enable on-site, real-time processing of the acquired spectra. This would allow water quality parameter estimates to be generated immediately after data acquisition, further improving operational efficiency and supporting near-real-time monitoring and decision making.

## 5. Conclusions

In this study, we developed and field-tested a ground-based hyperspectral remote sensing system (GHRSS) for continuous above-water spectral observation, which is characterized by a spectral resolution of 1 nm between 400 nm and 900 nm and a minimum sampling interval of 10 s. The system integrates radiometric acquisition, automated control, observation azimuth control, wireless data transmission, and solar-powered operation, enabling stable long-term deployment in dynamic aquatic environments. Through field deployment in the Cao’e River, the GHRSS successfully acquired high-frequency hyperspectral Rrs with reliable radiometric performance and quality control. Based on paired hyperspectral observations and in situ measurements, empirical and machine learning algorithms (PLSR and SVR) were developed to retrieve DOC and POC from the GHRSS based on sampled data covering different optical properties. SVR is the best algorithm for inverting DOC and POC with the GHRSS. Compared with in situ sampling, underwater sensors, and UAV and satellite remote sensing, the GHRSS can continuously provide high-frequency data and conserve labor and material resources with simple operation. The optical fibers of the GHRSS are less susceptible to contamination from substances in the water; thus, its operation and maintenance costs are low. The proposed system has an accurate radiation signal and a high signal-to-noise ratio. Short and rapid DOC or POC changes were observed within one day based on the GHRSS minute-scale monitoring, which highlighted the importance of high-frequency observation of DOC or POC. In the future, the GHRSS has strong potential to enhance our understanding of the dynamics and driving mechanisms of aquatic carbon in inland waters. It may also serve as an important component of integrated aquatic environmental monitoring systems.

## Figures and Tables

**Table 1:** Main optical and technical specifications of the GHRSS.

Category	Value/Description
Sensor type	Miniature fiber-optic spectrometers with crossed Czerny–Turner optical configuration
Fiber interface	SMA905 connector
Sensor weight	Approximately 0.7 kg per spectrometer
Spectral detection range	400–900 nm
Maximum spectral resolution	0.4 nm
Minimum integration time	10 μs
Minimum sampling interval	10 s
Data acquisition window	9:00–16:00 local time
Observation geometry	(40°, 135°)
Control unit	Microcontrol board with Win 10 system and i7 processor
Maximum power consumption of control board	Approximately 40 W
Data transmission	4G communication module with TCP/IP transmission and MQTT cloud backup
Power system	12 V battery combined with solar-panel charging

**Table 2:** Best-performing spectral indices identified from the correlation analysis for DOC and POC.

Target Parameters	Spectral Index	|r|
DOC	*R_rs_*(604)	0.75
	*R_rs_*(691)/*R_rs_*(484)	0.81
	*R_rs_*(592) + *R_rs_*(705)	0.76
	*R_rs_*(455) − *R_rs_*(456)	0.75
POC	*R_rs_*(819)	0.65
	*R_rs_*(838)/*R_rs_*(839)	0.69
	*R_rs_*(819) + *R_rs_*(820)	0.56
	*R_rs_*(832) − *R_rs_*(889)	0.71

**Table 3:** The R^2^, RMSE, MAE and RPD of different DOC and POC estimation models.

	Models	R^2^	RMSE (mg L^−1^)	MAE (mg L^−1^)	RPD
DOC	logarithmic	0.500	0.207	0.122	1.443
	PLSR	0.947	0.059	0.028	4.440
	SVR	0.979	0.031	0.024	6.997
POC	logarithmic	0.499	0.353	0.282	1.422
	PLSR	0.910	0.152	0.067	3.403
	SVR	0.960	0.152	0.066	3.417

## Data Availability

The data will be made available upon request. Researchers interested in accessing the data may contact the authors.

## Abbreviations

The following abbreviations are used in this manuscript:

DOC	Dissolved Organic Carbon
POC	Particulate Organic Carbon
GHRSS	Ground-Based Hyperspectral Remote Sensing System
*R_rs_*	Remote-Sensing Reflectance
*L_w_*	Water-Leaving Radiance
*L_wn_*	Normalized Water-Leaving Radiance
DNs	Digital Numbers
QC	Quality Control
CV	Coefficient of Variation
NDVI	Normalized Difference Vegetation Index
NDWI	Normalized Difference Water Index
HTCO	High-Temperature Catalytic Oxidation
PIC	Particulate Inorganic Carbon
PLSR	Partial Least Squares Regression
SVR	Support Vector Regression
RMSE	Root Mean Square Error
MAE	Mean Absolute Error
RPD	Relative Prediction Deviation
ASD	Analytical Spectral Device
MRE	Mean Relative Error
PD	Percent Difference
UAV	Unmanned Aerial Vehicle

## Appendix A

### Model Evaluation Metrics

The performance of the retrieval models was evaluated using four statistical metrics, including the coefficient of determination ($R^{2}$), root mean square error (*RMSE*), mean absolute error (*MAE*), and residual prediction deviation (*RPD*).

The coefficient of determination ($R^{2}$) was used to describe the proportion of variance in the measured values that could be explained by the model and was calculated as follows:

(A1) $$R^{2}=1-\frac{\sum_{i=1}^{n}{\left(y_{i}-{\hat{y}}_{i}\right)}^{2}}{\sum_{i=1}^{n}{\left(y_{i}-{\bar{y}}_{i}\right)}^{2}}$$

The root mean square error (*RMSE*) was used to quantify the overall prediction error of the model:

(A2) $$RMSE=\sqrt{\frac{1}{n}\overset{n}{\underset{i=1}{\sum }}{\left(y_{i}-{\hat{y}}_{i}\right)}^{2}}$$

The mean absolute error (*MAE*) was used to reflect the average magnitude of the absolute prediction error:

(A3) $$MAE=\frac{1}{n}\overset{n}{\underset{i=1}{\sum }}\left|y_{i}-{\hat{y}}_{i}\right|$$

The residual prediction deviation (*RPD*) was calculated as the ratio of the standard deviation of the measured values to the *RMSE*:

(A4) $$RPD=\frac{SD}{RMSE}$$

where $y_{i}$ and ${\hat{y}}_{i}$ are the measured and predicted values of the i-th sample, respectively, $\bar{y}$ is the mean of the measured values, n is the total number of samples, and SD is the standard deviation of the measured values. In general, better model performance is indicated by higher $R^{2}$ and *RPD* values and lower *RMSE* and *MAE* values.
